# Time-to-event analysis in economic evaluations: a comparison of modelling methods to assess the cost-effectiveness of transplanting a marginal quality kidney

**DOI:** 10.1186/s13561-021-00312-4

**Published:** 2021-04-15

**Authors:** Sameera Senanayake, Nicholas Graves, Helen Healy, Keshwar Baboolal, Adrian Barnett, Sanjeewa Kularatna

**Affiliations:** 1 Australian Centre for Health Services Innovation (AusHSI) and Centre for Healthcare Transformation, Queensland University of Technology (QUT), 60 Musk Ave, Kelvin Grove, QLD 4059 Australia; 2 Duke-NUS Medical School, 8 College road, Singapore, Singapore; 3 Royal Brisbane Hospital for Women, Brisbane, Australia; 4 School of Medicine, University of Queensland, Brisbane, Australia

**Keywords:** Survival analysis, DES model, Markov model, Kidney transplantation

## Abstract

**Background:**

Economic-evaluations using decision analytic models such as Markov-models (MM), and discrete-event-simulations (DES) are high value adds in allocating resources. The choice of modelling method is critical because an inappropriate model yields results that could lead to flawed decision making. The aim of this study was to compare cost-effectiveness when MM and DES were used to model results of transplanting a lower-quality kidney versus remaining waitlisted for a kidney.

**Methods:**

Cost-effectiveness was assessed using MM and DES. We used parametric survival models to estimate the time-dependent transition probabilities of MM and distribution of time-to-event in DES. MMs were simulated in 12 and 6 monthly cycles, out to five and 20-year time horizon.

**Results:**

DES model output had a close fit to the actual data. Irrespective of the modelling method, the cycle length of MM or the time horizon, transplanting a low-quality kidney as compared to remaining waitlisted was the dominant strategy. However, there were discrepancies in costs, effectiveness and net monetary benefit (NMB) among different modelling methods. The incremental NMB of the MM in the 6-months cycle lengths was a closer fit to the incremental NMB of the DES. The gap in the fit of the two cycle lengths to DES output reduced as the time horizon increased.

**Conclusion:**

Different modelling methods were unlikely to influence the decision to accept a lower quality kidney transplant or remain waitlisted on dialysis. Both models produced similar results when time-dependant transition probabilities are used, most notable with shorter cycle lengths and longer time-horizons.

**Supplementary Information:**

The online version contains supplementary material available at 10.1186/s13561-021-00312-4.

## Introduction

Decision makers in health services commonly make resource allocation choices, aiming to select for high value care. The processes of choice selection include arbitrary, eminence, experience or evidence based. Economic evaluations estimate changes in the costs to health benefits of competing health interventions, and so are an ideal process for informing resource allocation decisions. Economic evaluation of health interventions lends itself to the decision analytic approach, using models such as decision trees, Markov models, and discrete event simulations (DES) [1]. The choice among these models is a critical step in the process. Model selection is context specific, with deployment of inappropriate models yielding results that could be flawed/lower value decision making [2].

Donor kidney quality is an important factor that influences graft survival. Lower quality donor kidneys are associated with increased risks of graft failure, earlier return to dialysis and higher post-transplantation costs [3, 4]. The ever-increasing demand for donor kidneys is driving decision makers to consider lower quality kidneys, predicated on the premise that any transplant offers superior patient survival and quality of life compared to remaining on dialysis. Decision analytic models bring rigour to the premise, systematically assessing the relative cost/benefits of transplanting even a lower quality kidney compared with remaining on dialysis. Our review of the evidence of cost-utility in people with chronic kidney disease (CKD) undergoing transplant found Markov models are by far the most common decision analytic model used [5]. Axelrod et al. was the only publication we found deploying DES modelling [6]. They reported transplanting a lower quality kidney has an incremental cost-effectiveness ratio (ICER) of USD 32,870 per quality adjusted life years (QALY) compared with remaining on dialysis. The willingness to pay threshold (WTP) is a systems decision that varies between countries. Therefore, ICER of USD 32,870 can be either above or below the WTP threshold depending on the country of concerned.

However, when we deployed Markov models, our studies found that transplanting a lower quality kidney is the dominant strategy (cost saving and more effective) compared with remaining on dialysis [7]. These two studies cannot be comparable due to differences in data sources and methods deployed to estimate output products. A comparison between the two-different decision analytic models, Markov versus DES controlling for all other factors, is required to answer the question: does the choice of modelling method affect the outcome and hence impact policy and clinical decisions?

Markov models are best suited if repeated outcomes occur over time, such as in most chronic ie longitudinal diseases like CKD. The transition from one health state to another is based on transition probabilities [8]. Most often it is assumed that transition probabilities are constant over time, thus the same probability is used across all cycles of the model [2]. This is a likely over-simplification in CKD where clinical outcomes like mortality differ depending on the health state and time, leading to potentially misleading results [9]. For example, the probability of graft failure following kidney transplant reduces over time, whereas the probability of mortality while waitlisted for the kidney increases [10, 11]. Transition probabilities that increase or decrease over time are factored into the models by using transition probabilities specific for how long a person is in a health state.

Discrete event simulation models use patient level simulations to estimate the costs and benefits of health interventions across a patient population [12]. The models have the power to factor in random timing of events, in contrast to Markov models that assume events occur at regular intervals. They do this by modelling distributions for patient-level times-to-event [13] whereas Markov models use the same transition probabilities for all patients in the same health state. This means that a patient can experience an event at any time rather than at regular intervals as used by Markov models.

If patient information over time is available, as in a registry, time-dependent transition probabilities for a Markov model and time-to-event estimates for individual patients in a DES model can be estimated for time-to-event analysis eg survival analysis [9, 14]. Time-to-event analysis is superior in adjusting for censoring, a not uncommon feature of longitudinal data. Some survival models handle the missing data by extrapolation of model parameters beyond the duration of the censored data. This is particularly important when the time-horizon of the model is long, such as in lifetime time-horizon. However, of the different survival approaches available (e.g. Non-parametric, semi-parametric, parametric), the one selected to estimate the parameters of a decision analytic model (i.e. Markov or DES) substantially impacts the cost-effectiveness results [15]. Therefore, choice of the appropriate survival approach is an important variable to consider when performing time-to-event analysis in estimating output products of a decision analytic model. The best established survival approach in estimating output products of Markov and DES modelling is parametric [16, 17].

There is a lack of robust evidence on the cost-effectiveness of transplanting a low-quality kidney and the literature reports mixed results using DES and Markov models. In this study we therefore aim to deploy Markov and DES modelling to compare the cost-effectiveness of transplanting a lower-quality kidney compared with remaining waitlisted for a kidney on dialysis. Multiple published studies comparing the output products of Markov and DES models all used fixed transition probabilities for the Markov models. None assessed the impact of deploying a parametric survival approach into the input parameters. We deploy the parametric survival approach into the time-dependent transition probabilities of the Markov model and the distributions of time-to-event analysis in the DES model in this study.

## Methods

**Fig. 1:**
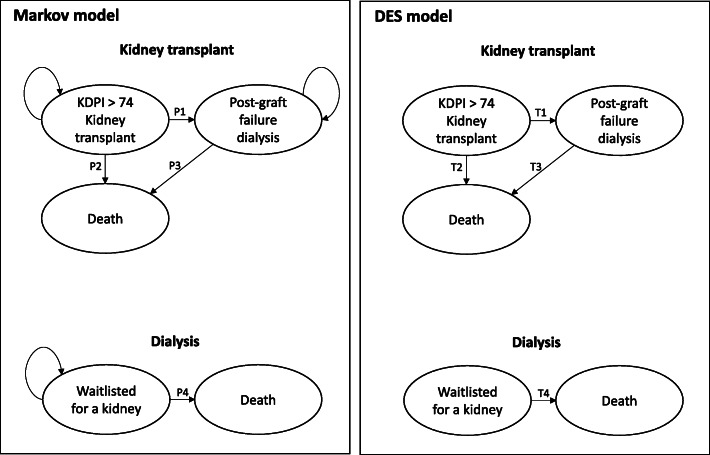
Markov and the Discrete Event Simulation models used in the analysis. P1: Probability of graft failure after transplantation; P2: Probability of death after transplantation; P3: Probability of death after graft failure; P4: Probability of death while waitlisted. T1: Time-to-graft failure after transplantation; T2: Time-to-death after transplantation; T3: Time-to-death after graft failure; T4: Time-to-death while waitlisted

Decision analytic models: a Markov model and a Discrete event simulation model were constructed using TreeAge Pro 2020 with the purpose of estimating the incremental costs and quality-adjusted life years (QALY) (Fig. 1). Both models estimated the cost-effectiveness of transplanting a low-quality kidney as compared with remaining waitlisted for a kidney. People who remain waitlisted will be on dialysis throughout the time horizon of the study. The quality of a donor kidney was defined using the Kidney Donor Profile Index (KDPI), which ranges from 0 to 100%, with higher scores indicating lower donor quality [18]. A kidney with a KDPI more than 74 was defined as a lower quality kidney. The decision analytic models were validated using the method proposed by Vemer et al. (2016) (Supplementary material; Table 1) and found that the models have adequate validity.

### Target population

The target population for the economic evaluation was 50-year old chronic kidney disease patients undergoing kidney transplant in Australia.

### Markov models

In a Markov model, a cohort of patients moves from one health state to another at defined times depending on the assigned transition probabilities. We used time-dependent transition probabilities for the Markov model derived from cumulative survival distributions, meaning that the transition probabilities change as the time in a health state increases. This is discussed in more details in the section on model parameters.

The Markov model developed for transplanting a low-quality kidney (KDPI > 74) had three health states: transplantation, return to dialysis post kidney transplantation failure and death (Fig. 1). The cohort starts at “KDPI > 74 kidney transplant” health state and tracks the outcomes of: return to dialysis post kidney transplantation failure, death, or successfully functioning transplant. In the event of graft failure, the patient can have subsequent outcomes of either remain on dialysis or die while on dialysis. It was assumed that no patients had a re-transplantation following graft failure. The Markov model for the cohort remaining waitlisted for a kidney had two health states: die while on dialysis or remain healthy (Fig. 1).

We fitted four Markov models (Table 1) with cycles of 6 or 12 months, each with two-time horizons (5 and 20 years). Different time-horizons were used to assess how the main outputs change over time, while different cycle lengths were used to assess whether Markov models with shorter cycle length would more closely correspond with the DES model results. A half-cycle correction was used for all the models.

**Table 1:** Markov models fitted in the study

Model	Cycle (years)	Time horizon (years)
1	0.5	5
2	0.5	20
3	1	5
4	1	20

### DES models

In a DES model, individual patients undergo a series of processes (events) affecting the outcomes, costs and quality adjusted life years (QALYs) over time [2], experiencing events in continuous time. These continuous times are sampled for individual patients from probability distributions, the parameters of which are selected to create distributions that match observed data on survival times.

All patients transplanted with low-quality kidney (KDPI > 74) would experience either returning to dialysis post kidney transplantation failure or death as their next event. When the patient is on dialysis post graft failure, the next event they could experience is death. The only next event the patients who are waitlisted could experience is death (Fig. 1). The time spent in each health state is directly drawn per patient from the corresponding cumulative survival distribution (see below).

### Data sources

#### Cost data

Initial and follow-up costs for a kidney transplant were extracted from the report, “The economic impact of end-stage kidney disease in Australia - Projections to 2020” by Kidney Health Australia in 2010 [19]. The estimated cost of a deceased donor kidney transplant in the first year of surgery was AUD 81,549 (2010), which included surgery and hospitalisation costs, immunosuppressive therapy, specialist review and consultations, drugs and donor costs for a transplant. The cost of annual follow-up care (from the second year onwards) was estimated to be AUD 11,770 (2010), which included immunosuppressive therapy, drugs and non-drug follow-up costs. However, the costs presented in the above study represented an average cost for transplants of different donor kidney quality levels (both high and low quality). Compared with a high-quality donor kidney transplant, a low-quality donor kidney transplant is expected to incur higher costs due to greater likelihood of high short-term costs from events such as delayed graft function and increased drug costs [20, 21]. Therefore, using expert opinion, a conservative 15% increase was allocated for a KDPI > 74 kidney transplant.

The costs for a dialysis patient were according to the New South Wales Dialysis Costing Study (2008), which reported procedural and non-procedural costs of managing chronic kidney disease [22]. The annual cost for a dialysis patient was calculated as a blend of in-centre haemodialysis, satellite-centre haemodialysis, home haemodialysis and peritoneal dialysis, in proportion to current usage patterns of the different dialysis modalities (AUD 69,089). All costs were converted to 2020 Australian dollars [23].

#### Utility data

Utility values among kidney transplant patients and dialysis patients were sourced from a 2012 systematic review and meta-analysis [24]. The utility score among transplant patients was estimated to be 0.82 (95% CI 0.74 to 0.90), while among dialysis patients, it was estimated to be 0.70 (95% CI 0.62 to 0.78), without differentiating between different dialysis modalities.

#### Parameter estimation for both Markov and DES models

Model parameters for both Markov and DES models were estimated from data sourced from the Australia and New Zealand Dialysis and Transplant Registry (ANZDATA). ANZDATA collects and reports the incidence, prevalence and outcome of dialysis treatment and kidney transplantation for patients with end stage kidney disease across Australia and New Zealand. Strengths of the Registry are the completeness of data captured across Australia and New Zealand, longevity of follow-up information and accessibility [25]. Model parameters for the different health states were estimated in three groups of patients:

1. Patients who had a KDPI > 74 donor kidney transplantation between 1st January 2007 and 31st December 2017 in Australia.
2. Patients who started dialysis of any modality between 1st January 2007 and 31st December 2016 in Australia
3. Patients who have ever been waitlisted for a kidney transplantation between 1st January 2007 and 31st December 2017 in Australia but never had a kidney transplantation.

The parameters for both models were calculated from time-to-event analysis (survival analysis). Four parametric distributions were fitted to the data: Exponential, Weibull, Log-logistic and Log-normal. Latimer recommended fitting more than one parametric model and then selecting the best based on statistical and visual fit [15]. The best model was selected based on its statistical fit (using the Akaike and Bayesian information criterion (AIC, BIC) [26]) to the observed data. The best fitting model was further justified using its visual fit to the observed data [27]. Weibull regression had the best (lowest) AIC and BIC (Table 2). Its visual fit to observed data further confirmed the appropriateness of the Weibull model. Therefore, Weibull regression was used to estimate the time-dependent transition probabilities for the Markov model, and time-to-event parameters for the DES model. The Lambda (λ–rate parameter) and Gamma (ϒ–shape parameter) were used to calculate the time-dependent transition probabilities in the Markov model according to the method described by Briggs et al. [9], whereas the same parameters (Lambda and Gamma) were used to sample the time-to-event estimates for each individual patient in the DES model (Table 3).

**Table 2:** Comparison of AIC and BIC values using Cox, Exponential, Weibull and Log-logistic regression methods

Parameter	*Akaike information criterion (AIC)*				*Bayesian information criterion (BIC)*			
	Expo	Weibull	Log-L	Log-N	Expo	Weibull	Log-L	Log-N
Graft failure following transplantation	1060.2	896.1	898.4	909.0	1065.2	906.1	908.4	919.0
Mortality following transplantation	1628.7	1628.1	1635.5	1666.2	1638.7	1633.1	1645.4	1676.2
Mortality following graft failure	23,759.2	23,700.6	23,770.5	24,046.3	23,766.3	23,714.8	23,784.7	24,060.5
Mortality while waitlisted	2093.2	2042.6	2047.9	2086.6	2098.4	2053.1	2058.4	2097.1

*Expo*: *Exponential model; Log-L: Log-logistic model; Log-N: Log-normal model*

**Table 3:** Parameter estimates and uncertainties used in the models

Parameter		Baseline value	Standard Error	Distribution	Source
**Transition probabilities**					
Graft failure following transplantation	Lambda	0.0698	0.0072	Normal	ANZDATA
	Gamma	0.3944	0.0345	Normal	ANZDATA
Mortality following transplantation	Lambda	0.0502	0.0059	Normal	ANZDATA
	Gamma	0.9305	0.0572	Normal	ANZDATA
Mortality following graft failure	Lambda	0.0922	0.0027	Normal	ANZDATA
	Gamma	1.1161	0.0153	Normal	ANZDATA
Mortality while on waiting list	Lambda	0.0315	0.0039	Normal	ANZDATA
	Gamma	1.4346	0.0654	Normal	ANZDATA
**Utility**					
Transplant		0.82; 95% CI (0.74 to 0.90)		Uniform	[24]
Dialysis		0.70; 95% CI (0.62 to 0.78)		Uniform	[24]
**Cost (in AUD)**					
Transplant (1st year)		115,725 (± 15%)		Uniform	[19]
Transplant (2nd year onwards)		16,110 (± 15%)		Uniform	[19]
Dialysis		81,689 (± 15%)		Uniform	[22]

The following time-dependent transition probabilities (Markov model) and time-to-event parameters (DES model) were estimated:

1. Graft failure following transplanting a KDPI > 74 donor kidney
2. Mortality following transplanting a KDPI > 74 donor kidney
3. Mortality following graft failure
4. Mortality while on waitlist for a kidney (only waitlisted patients were included)

Both decision analytic models were simulated for two-time horizons (5 years and 20 years) to assess how the main outputs of the two models change over time.

#### Model evaluation

The main outputs of both Markov and DES models were expected costs, expected QALY and Net Monetary Benefit (NMB) for a patient who underwent a low quality (KDPI > 74) kidney transplantation and a patient on dialysis who is waitlisted for a donor kidney. We used a Willingness To Pay (WTP) value of AUD 28,000, which reflects the opportunity cost of additional healthcare expenditures within a constrained budgetary environment [28].

DES models are stochastic, and so produce different results when run with different or even same number of patients [13]. Hence, the model was run multiple times with varying number of patients to determine the appropriate size of the patient population that would produce stable NMB [29].

Parameter uncertainty was determined by probabilistic sensitivity analyses using Monte Carlo simulation. Both Markov and DES models were simulated for 5000 iterations and, during each iteration, the model inputs were sampled from the fitted distributions (Table 2). The results of probabilistic sensitivity analyses were summarized using NMB and were calculated using the following formula:

$$ NMB=\left( WTP\times \kern0.5em QALY\right)- Costs $$

The highest NMB is the most cost-effective decision and choosing anything else incurs an opportunity cost.

Both QALYs and costs were discounted at an annual rate of 5% as recommended by the Medical Services Advisory Committee’s Technical Guidelines, Australia [30]. The perspective of the analyses was that of the healthcare payer.

## Results

**Fig. 2:**
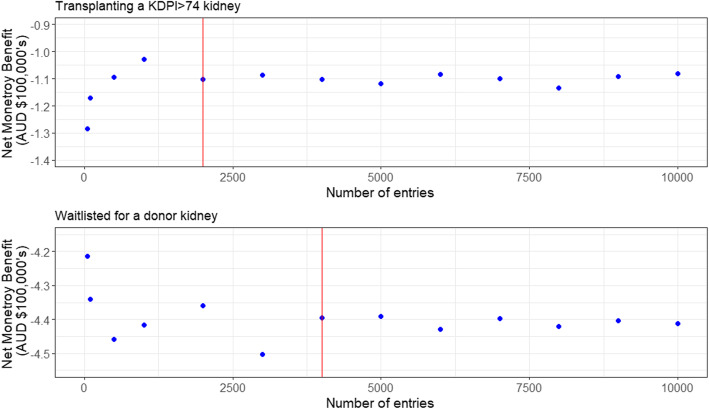
Net monetary benefits produced from DES model with varying number of simulated patients. *The red vertical line indicates the population size where the net monetary benefit stabilized*

NMB from DES model with varying number of simulated patients are in Fig. 2. The estimated NMB of the DES model transplanting a KDPI> 74 kidney stabilized around AUD − 110,000 for a patient population size of 2000. The NMB of waiting for a donor kidney stabilized only after 4000 patients. Therefore, the results of DES are from simulations of 4000 patients.

In the base case analysis, irrespective of the modelling method (Markov or DES), the cycle length of Markov model (6 or 12-months) or the time horizon (5 and 20 years) transplanting a low-quality kidney was both cost saving and more effective (i.e. the dominant strategy) compared to remaining waitlisted while on dialysis. Of the three models (6 and 12 month cycle length Markov models and the DES model), the Markov model with 12 month cycle lengths recorded the highest cost and the highest QALY for “Waitlisted for a kidney” strategy. The DES model recorded the highest total cost for “Transplanting a KDPI>74 kidney”, while the Markov model with six-month cycle lengths had the highest total QALY. This was true for both of the two-time horizons (Table 4).

**Table 4:** Cost (AUD), QALY and net monetary benefit per patient by model and time-horizon for the Markov and DES models

		Base case analysis					Probabilistic sensitivity analysis			
		Cost^**#**^	Effect	Δ Cost^**#**^	Δ Effect	ICER	Net monetary benefit (NMB)		Δ Net monetary benefit	
							Mean NMB^**#**^	% change from the DES value	Δ NMB^**#**^	% change from the DES value
**Time horizon – 5 years**										
Markov model (cycle length 1 year)	Transplanting KDPI > 75 kidney	147,000	3.17	196,000	0.35	Dominant	−49,000	142%	209,000	−33%
	Waitlisted for a kidney	343,000	2.82				− 258,000	0.3%		
Markov model (cycle length 0.5 year)	Transplanting KDPI > 75 kidney	142,000	3.20	187,000	0.50	Dominant	−96,000	22%	191,000	−26%
	Waitlisted for a kidney	328,000	2.70				− 287,000	−10%		
Discrete Event Simulation model	Transplanting KDPI > 75 kidney	206,000	3.20	130,000	0.45	Dominant	−118,000		141,000	
	Waitlisted for a kidney	336,000	2.75				− 259,000			
**Time horizon – 20 years**										
Markov model (cycle length 1 year)	Transplanting KDPI > 75 kidney	267,000	6.89	371,000	1.64	Dominant	−53,000	128%	416,000	−17%
	Waitlisted for a kidney	639,000	5.25				− 469,000	−0.7%		
Markov model (cycle length 0.5 year)	Transplanting KDPI > 75 kidney	248,000	7.17	299,000	2.68	Dominant	− 146,769	−17%	329,000	5%
	Waitlisted for a kidney	547,000	4.49				− 476,000	−2%		
Discrete Event Simulation model	Transplanting KDPI > 75 kidney	322,000	7.09	285,000	2.09	Dominant	− 121,000		345,000	
	Waitlisted for a kidney	607,000	5.00				− 466,000			

^*#*^ *Rounded up to the nearest AUD 1000*

Results of probabilistic sensitivity analysis indicate that transplanting a lower quality kidney results in the highest NMB compared to waitlisted for a kidney in all models. Compared with the NMB of transplanting a lower quality kidney in a DES model, transplanting a lower quality kidney in the Markov model with 12 month cycle lengths is nearly 150% higher. This difference reduced to around 20% when the results were compared with the Markov model with six-month cycle lengths. However, in the “Waitlisted for a kidney” strategy, which had only two health states in the Markov models (one event in the DES model), the difference between the NMB of DES and the two Markov models was around 10% in both of the two-time horizons. (The difference was < 1% in the Markov model with 12 month cycle lengths). The incremental NMB of a Markov model with six-month cycle lengths was closer to the incremental NMB of the DES model than the Markov model with 12 month cycle lengths. Furthermore, this gap reduces from 27 to 6% as the time horizon increased from 5 to 20 years.

**Fig. 3:**
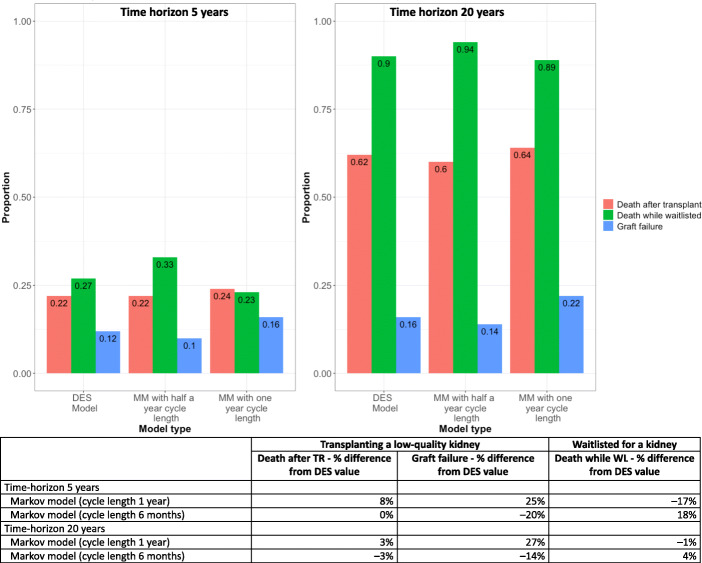
Proportion of events predicted by Markov and DES models over the two time horizons and the percentage difference from the DES value

The differences in costs and QALYs observed in the three models may be due to differences in numbers of graft failures or of patients dying. The proportion of events generated from the three models during the two-time horizons is in Fig. 3. In both of the two-time horizons, Markov models with 12 month cycle lengths had the highest proportion of graft failures and highest proportion of deaths after transplantation. The highest proportion of deaths while waitlisted was in the Markov model with six-month cycle lengths. The relative differences in deaths after transplantation and deaths while waitlisted in the latter model compared to the DES model decreased as the time horizon increases. However, graft failures after transplantation showed variable results.

The Markovian assumption means Markov models do not account for long-term memory, thus the total time a patient spent in a health state cannot be estimated [31]. However DES models do not have this limitation and model observed time-to-event. Figure 3 shows the density plots of time to death after transplantation, time to graft failure after transplantation and time to death while waitlisted, from DES modelling and actual data at the 5-year time horizon. Plots of time to death after transplantation and time to graft failure after transplantation modelled using DES have a close resemblance to the actual data. However, the DES model predicted patients to have longer times to death while waitlisted as compared with the actual data.

## Discussion

This study compared cost-effectiveness when different types of modelling techniques (Markov and DES) were used to model outcomes of transplanting a lower-quality kidney versus remaining waitlisted for a kidney. We used parametric survival models to estimate the time-dependent transition probabilities of Markov models and distribution of time-to-event in DES models. To the authors knowledge, this is the first cost-effectiveness study to compare the results when Markov and DES are modelled with parameters estimated from parametric survival methods, for any disease condition. Both models were developed from the same patient datasets. DES model output had the closer resemblance to the actual data, thus, we are of the view that this modelling method gives a closer approximation to reality. Our evaluation found that transplanting a low-quality kidney was both cost saving and more effective (i.e. the dominant strategy) as compared to remaining waitlisted while on dialysis. The finding was irrespective of the modelling method, cycle lengths of Markov model or the time horizon. The differences of incremental NMB between Markov model and DES model reduces with the shorter cycle lengths and longer time horizons.

The choice of the survival model used to estimate the parameters of decision analytic models alter the cost-effectiveness results, and thus may affect planning and policy decisions [15, 32]. The semi-parametric Cox proportional hazard method is the most commonly used survival model in the literature [33]. However, its utility in estimating time-dependent probabilities in Markov models is limited by its inability to model how the risk of an event changes over time [9]. Furthermore, the Cox model does not allow extrapolation beyond the last follow-up time which limits its use in health economic modelling [31]. Another option would be estimating the transition probabilities directly from published Kaplan–Meier curves, which is a non-parametric method [17]. However, Kaplan–Meier curves tend to over fit the empirical data, which impacts generalisability of the estimated transition probabilities [34]. The current study used parametric survival methods, with the power of modelling time-dependency and extrapolate to longer times outside the observed data. Parametric survival methods are best suited for our research question of looking at long-term outcomes.

The literature on which of cohort-based Markov models and DES models is superior is inconclusive. Some studies show that the results are not substantially different between the two modelling approaches [35], while others demonstrate sizable differences [36, 37]. However, it is important to note that in these comparisons, Markov models used fixed transition probabilities whereas, in the current study, we used time-dependant transition probabilities. Therefore, the results of the current study add knew knowledge to the continuing debate about the superiority of different decision analytic methods [38]. Our results indicate, for our research question, different modelling methods are unlikely to influence the decision which produces best value for money. However, costs and effectiveness measures (QALY) differed between the two models. The Markov model with the longer cycle lengths recorded the highest cost and the highest QALY for “Waitlisted for a kidney” strategy. The DES model recorded the highest cost for “Transplanting a KDPI>74 kidney”, while the Markov model with the shorter cycle lengths had the highest QALY. Though these discrepancies may not affect the direction of the funding decision, they may influence subsequent analyses such as budget impact analysis [39].

**Fig. 4:**
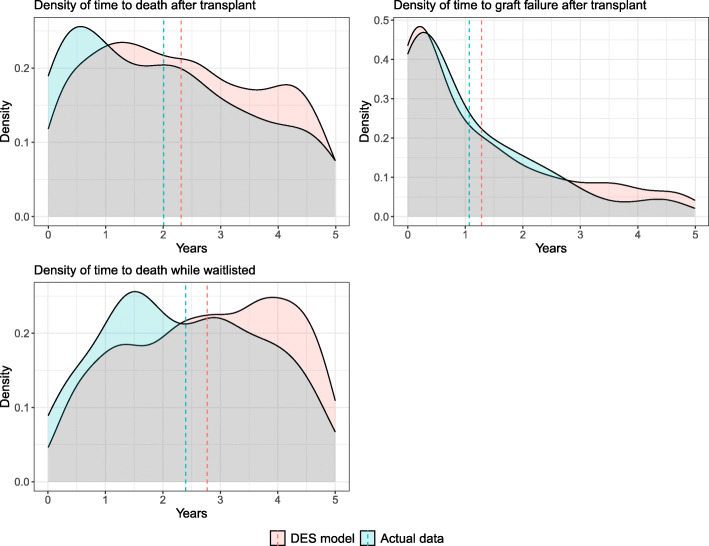
Density plots of time to death after transplantation, time to graft failure after transplantation and time to death while waitlisted from DES model (single simulation) and actual data. The dotted vertical lines are the mean for the model and data

In the DES model, the predicted time-to-event for each of the simulated patients was a function of the parametric survival method. This method ensures that the times-to-event could vary even for two identical patients. This random variability is often seen in clinical practice, thus, results generated from the DES model have the potential to more closely represent the variability seen in real-life. This was evident in the density plots presented in Fig. 4, where predictions from a DES model closely approximated the actual data.

Net monetary benefit and incremental net monetary benefit were the main outputs of this cost-effective analysis. Of the two Markov models, Markov models with shorter cycle lengths came close to DES results. Difference between the models got smaller as the time horizon increased, with almost similar incremental net monetary benefit (6% difference) when the time horizon was 20 years. Almost identical prediction of events (i.e. death after transplantation, graft failure after transplantation and death while waitlisted) by the Markov model with shorter cycle lengths and DES model at 20 year time horizon may explain the finding (see Figure 3). Furthermore, in the DES model, events can occur at any time, whereas in Markov events occur at fixed intervals (i.e. cycle lengths). Therefore, Markov models could have artificial time delays of events occurring, compared to DES models and real-life [37]. The Markov models with shorter cycle lengths have less delays, which could explain the narrow difference of the main outputs between Markov with six-month cycles and DES model. Several authors have previously discussed the possibility of using shorter cycle lengths to reduce the gap between Markov and DES [2, 40], and we were able to demonstrate this with empirical data.

Debate exists regarding the choice of the model structure. Some authors argue that DES should always be the preferred modelling method for economic evaluations [38, 41]. Others authors highlighted situations where a DES model is preferred [42]. DES models capture the heterogeneity of the patient population, an important feature of chronic kidney disease patient populations. Therefore, a DES model seems more appropriate to address the research question of the current study. However, we demonstrated that use of shorter cycle lengths and time-dependant transition probabilities in a Markov model produces comparable results to a DES model. The Markov model used in the present study had only three health states and further research is needed to assess whether complex Markov models (multiple health states), with shorter cycle lengths and time-dependant transition probabilities would produce results similar to DES models.

This study has limitations. First, the decision analytic models did not account for the clinical health state of re-transplants following graft failure, as the scope of the study was to assess the costs and outcomes following the first kidney transplantation. However, the annual re-transplantation rate in Australia is around 3%, thus, authors believe this limitation would not have significantly changed the current results. Secondly, quality of the donor kidney was defined using KDPI, which has only reasonable discriminatory power of predicting graft failure. However, KDPI is the best available index to describe donor kidney quality and has previously been used to define marginal quality kidneys in economic evaluations [5, 6, 43]. Finally, we adopted a healthcare-payer perspective which does not capture costs incurred by the patient. Analysis from a societal perspective, which includes patient costs, could affect the cost-effectiveness results.

## Conclusion

This study found that although Markov and DES models produced different outcomes, both delivered the same conclusion that transplanting even a low-quality kidney is cost-effective compared to remaining on dialysis. The finding is robust, with similar outputs from the Markov and DES models as the conditions of the time-dependant transition probabilities ie shorter cycle lengths and longer time-horizons were modelled.

## Supplementary Information

**Additional file 1: Table S1.** Model validation according to Assessment of the Validation Status of Health-Economic decision models (AdViSHE).

## Data Availability

The datasets generated during and/or analysed during the current study are available from the corresponding author on reasonable request.

## Abbreviations

AIC: Akaike and Bayesian information criterion
ANZDATA: Australia and New Zealand Dialysis and Transplant Registry
BIC: Akaike and Bayesian information criterion
CKD: Chronic kidney disease
DES: Discrete-event-simulations
ICER: Incremental cost-effectiveness ratio
KDPI: Kidney Donor Profile Index
MM: Markov-models
NMB: Net monetary benefit
QALY: Quality adjusted life years
WTP: Willingness to Pay
