# Supplementary material for: Time-to-event analysis in economic evaluations: a comparison of modelling methods to assess the cost-effectiveness of transplanting a marginal quality kidney
Source: Health Econ Rev. 2021 Apr 15;11:13. doi: 10.1186/s13561-021-00312-4 (PMC8051030; doi:10.1186/s13561-021-00312-4)
Supplement: Supplementary file 1 — Additional file 1: Table S1. Model validation according to Assessment of the Validation Status of Health-Economic decision models (AdViSHE). [file 13561_2021_312_MOESM1_ESM.docx]

**Supplementary material**

**Table S1:** Model validation according to Assessment of the Validation Status of Health-Economic decision models (AdViSHE)

| **No** | **Criteria** | **Comment** |
| --- | --- | --- |
| *Part A: Validation of the conceptual model* | | |
| 1 | Face validity testing (conceptual model) : *Have experts been asked to judge the appropriateness of the conceptual model?* | The structural validity of the decision analytic models was assessed by an expert panel consisting of;   - two experienced nephrologists who manage patients with kidney transplants - three health economists who are experienced in developing decision analytic models - one statistician   The panel was of the view that the decision analytic models used in the study were appropriate to answer the research question. |
| 2 | Cross validity testing (conceptual model): *Has this model been compared to other conceptual models found in the literature?* | Prior to the study, the research team conducted the systematic review, *“Cost-utility analysis in chronic kidney disease patients undergoing kidney transplant; what pays? A systematic review”.* It reviewed the different decision analytic models that have been developed for kidney transplantation and the decision analytic models used in the current study were based on the results of the review. |
|  |  |  |
| *Part B: Input data validation* | | |
| 1 | Face validity testing (input data): *Have experts been asked to judge the appropriateness of the input data?* | The same expert panel mentioned in part A, assessed the face validity of the input data. |
| 2 | Model fit testing: *When input parameters are based on regression models, have statistical tests been performed?* | The transition probabilities were calculated using parametric survival models (i.e. Weibull regression) and necessary statistical tests were conducted to assess the suitability of the regression models. |
|  |  |  |
| *Part C: Validation of the computerized model* | | |
| 1 | External review: *Has the computerized model been examined by modelling experts?* | The models were developed in TreeAge pro 2020 software. The decision analytic models developed in TreeAge pro were validated by two experienced independent health economists who are experienced in developing models in TreeAge pro. |
| 2 | Extreme value testing: *Has the model been run for specific, extreme sets of parameter values in order to detect any coding errors?* | The results were consistent across different sets of extreme parameter values |
| 3 | Testing of traces: *Have patients been tracked through the model to determine whether its logic is correct?* | In the current study, the Markov models used cohort level simulations, while the discreet event simulations used patient level simulations. Both cohorts (Markov models) and patients (discreet event simulations) were tracked through the model to determine whether their logic is correct. |
|  |  |  |
| *Part D: Operational validation* | | |
| 1 | Face validity testing (model outcomes): *Have experts been asked to judge the appropriateness of the model outcomes?* | The same expert panel mentioned in part A, assessed the face validity of the results |
| 2 | Cross validation testing (model outcomes): *Have the model outcomes been compared to the outcomes of other models that address similar problems?* | The results were compared with 2 similar papers ^(1,2)^ and found that the results were compatible with the current evidence  *^1^Senanayake S, Graves N, Healy H, Baboolal K, Barnett A, Sypek MP, Kularatna S. Donor Kidney Quality and Transplant Outcome: An Economic Evaluation of Contemporary Practice. Value in Health. 2020 Dec 1;23(12):1561-9.*  *^2^Axelrod DA, Schnitzler MA, Xiao H, Irish W, Tuttle‐Newhall E, Chang SH, Kasiske BL, Alhamad T, Lentine KL. An economic assessment of contemporary kidney transplant practice. American Journal of Transplantation. 2018 May;18(5):1168-76.* |
| 3 | Validation against outcomes using alternative input data: *Have the model outcomes been compared to the outcomes obtained when using alternative input data?* | No. |
| 4 | Validation against empirical data: *Have the model outcomes been compared to empirical data?* | No |
